# Vulvar leiomyoma: a case report

**DOI:** 10.11604/pamj.2019.32.208.18480

**Published:** 2019-04-29

**Authors:** Safaa Ammouri, Chourouk Elkarkri, Najia Zeraidi, Amina Lakhdar, Abdelaziz Baydada

**Affiliations:** 1 Service de Gynécologie-Obstétrique et Endoscopie Gynécologique, Maternité Souissi, Faculté de Médecine et Pharmacie, Université Mohammed V, Rabat, Morocco

**Keywords:** Leiomyoma, vulva, large lip

## Abstract

Leiomyomas represent about 3.8% of all benign soft tissue tumors. Vulvar localization is very rare. We present a case of a vulvar leiomyoma and discuss diagnostic and therapeutic features of this disease. A 30-year-old female patient with no medical history, had a 5cm mass located in the left large lip causing a discomfort at the perineum especially in sitting and walking. She underwent a complete surgical excision of the mass. The pathological examination confirmed the diagnosis of a leiomyoma. There was no recurrence after 24-months' follow-up. The vulvar leiomyoma is a rare benign tumor. The diagnosis is made only postoperatively after resection of the mass. The treatment is essentially based on total excision of the mass with a good prognosis.

## Introduction

Leiomyomas represent about 3.8% of all benign soft tissue tumors [1]. They can develop anywhere in the body where smooth muscle is present. The most common site is the uterus [2]. Those of the vulva are particularly rare and can be confusing with Bartholin's cysts. In this article, we present a case of a vulvar leiomyoma and discuss diagnostic and therapeutic features of this disease.

## Patient and observation

**Figure 1:**
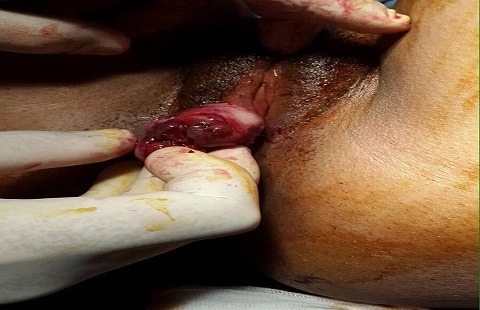
Aspect of the vulva mass at clinical examination

**Figure 2:**
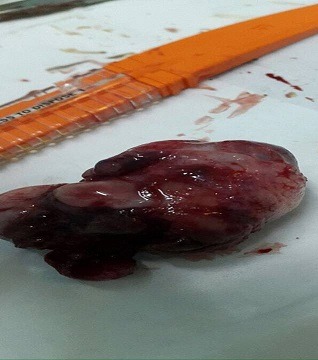
Specimen of the vulva mass measuring 4.5 x 3cm

**Figure 3:**
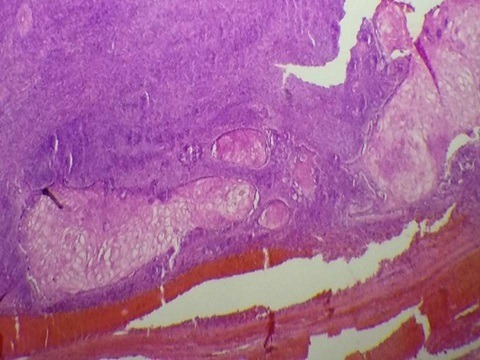
Squamous coat with tumor proliferation in crisscross beams HE x 4

**Figure 4:**
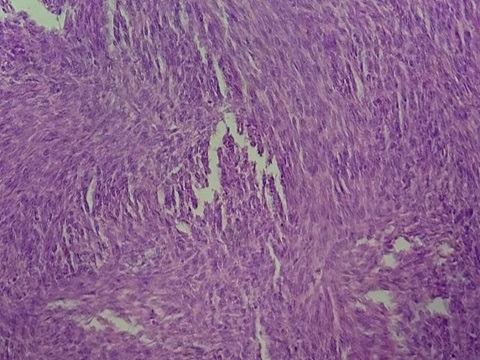
Well limited proliferation by a fibrous capsule and formed of bundles of cells with smooth muscular differentiation HER x 10

A 30-year-old female unmarried patient, with no medical history, having had menars at the age of 13 years, with regular cycles, had consulted for a vulvar mass which appeared recently since 3 months and increasing gradually in volume and causing a discomfort at the perineum especially in sitting and walking. On vulvar examination, there was a firm multilobulate pedicled mass measuring 5cm located at the posterior surface of the left large lip (Figure 1). The suprapubic pelvic ultrasound did not reveal intrauterine fibroid. The patient had a surgical excision of the vulva mass under local anesthesia (Figure 2). Microscopic examination revealed a proliferation of smooth muscle fibers which were gathered in intertwined bundles and separated by fibrosis and congestive vessels. The muscle fibers had a regular elongate nucleus with fine chromatin without atypical mitosis. The peripheral fibrous capsule was respected. Morphological analysis concluded to a vulvar leiomyoma (Figure 3, Figure 4). There was no recurrence after 24-months' follow-up.

## Discussion

Leiomyomas are frequent benign monoclonal tumors developed from smooth muscle cells [3]. Their etiology is still poorly understood, but it is likely that estrogens and progesterone are involved in tumor proliferation, as long as fibroids rarely appear before menarche and often regress after menopause [4]. Immunohistochemical analysis-when performed-reveals estrogen and/or progesterone receptors [1]. Several ectopic localizations have been reported in the literature: vulva, ovaries, urethra, bladder, peritoneum and retroperitoneum [5]. Vulvar leiomyomas are particularly rare. To our knowledge, only 160 cases have been reported in the literature [1]. Reidel *et al* found a single fibroid after examining 144 vulvar tumors [6]. Preoperative diagnosis can be difficult because of the rarity of this tumor and the non-specific clinical presentation. In preoperative, it is confused with the cyst of Bartholin or even a bartholinitis [1]. Clinical presentation varies. Nielsen *et al*. reported 25 cases of fibroids of the vulva. Most cases presented a painless mass. But sometimes there were symptoms of pain, itching, erythema [7]. Surgical monobloc excision of the mass is the main pillar of the treatment.

## Conclusion

The vulvar leiomyoma is a rare benign tumor. The diagnosis is often made only postoperatively after resection of the mass. Several hypotheses have been put forward to explain their origin, but the exact etiology remains unidentified. The treatment is essentially based on total excision of the mass.

## Competing interests

The authors declare no competing interests.
